# Submassive Pulmonary Embolism in the Setting of Intracerebral Hemorrhage: A Case of Suction Thrombectomy

**DOI:** 10.7759/cureus.32432

**Published:** 2022-12-12

**Authors:** William Ciurylo

**Affiliations:** 1 Internal Medicine, HCA/Tufts Medical School Portsmouth Regional Hospital, Portsmouth, USA

**Keywords:** thrombosis, thrombectomy, intracerebral hemorrhage, stroke, submassive, pulmonary embolism

## Abstract

Pulmonary embolism (PE) in the setting of intracerebral hemorrhage (ICH) is an unfortunate, challenging, and highly morbid clinical problem. Interventional strategies have lower associated bleeding risks than the standby for PE treatment: systemic anticoagulation. Despite this benefit, there are few examples in the literature of its utilization in the management of PE in the setting of ICH. This present case provides an example of the successful utilization of suction thrombectomy to manage PE in the setting of ICH.

An 80-year-old female presented to an outside hospital with complaints of dizziness, headache, nausea, and vomiting of abrupt onset one hour before arrival. Computed tomography (CT) of the head with CT Angiography (CTA) of the head and neck was performed and demonstrated hemorrhage in all ventricles; most prominently within the left lateral ventricle. Magnetic Resonance Imaging (MRI) of the brain suggested that the cause of her hemorrhage was reperfusion injury after a small acute infarction in the left internal capsule in the setting of anticoagulant use. Ten days after her diagnosis of ICH, a submassive PE was diagnosed with a class IV pulmonary embolism severity index (PESI). An interdisciplinary evaluation was conducted between hospitalist medicine, neurology, neurosurgery, and interventional radiology. A successful suction thrombectomy was performed on hospital day 11. No new neurologic deficits were appreciated post-procedure. The patient’s heart rate remained elevated but improved. Blood pressure remained controlled. The patient was weaned off oxygen to room air. Neurosurgery assessed the patient to be of acceptable risk for discharge with the further deferment of anticoagulation until repeat CT head six weeks after discharge. The patient was discharged on hospital day 14.

Treating PE in the setting of ICH is without clear guidelines. The appropriate treatment modality is reliant upon the clinical judgment and the individual details of each case. In this case, a high PESI with imaging demonstrating a stable hematoma without evidence of new blood resulted in the decision to use a suction thrombectomy. More research is needed to develop consistent evidence-based guidelines for this clinical challenge.

## Introduction

Pulmonary embolism (PE) in the setting of intracerebral hemorrhage (ICH) is an unfortunate and challenging clinical problem. The prevalence of PE in patients afflicted with ICH may be as high as 2% [1]. It is a potentially fatal combination with the mortality rate for all types of venous thromboembolism (VTE) in association with ICH being 35% to 52% [2]. The Oxford Community Stroke Project found that 5% of ICH patients died from PE within the first 30 days of ICH diagnosis [3]. Systemic anticoagulation is the treatment of choice for pulmonary embolism with a demonstrable reduction in morbidity and mortality [4]. But this treatment modality confers the risk of ICH expansion or recurrent bleeding that further the risks of disability and death [5]. As such, therapeutic anticoagulation is absolutely contraindicated within the first two weeks of an acute ICH [6,7]. In cases of atrial fibrillation and stroke prophylaxis dosed anticoagulation, restarting treatment after ICH is recommended as far as seven to eight weeks from the bleeding event [8].

A pulmonary embolism may not afford patients the ability to wait weeks for the initiation of treatment. Massive PE can lead to hemodynamic compromise, shock, and death, necessitating rapid intervention [9]. A 2018 case report described the dilemma of treating a massive PE in the setting of ICH [10]. In the absence of ICH, systemic thrombolysis, typically with tissue-type plasminogen activator (tPA), is indicated [11]. But systemic thrombolysis is in itself a risk factor for ICH [12]. The clinical team elected to forgo tPA; treating with an unfractionated heparin infusion followed by rivaroxaban, a direct-acting oral anticoagulant (DOAC). The outcome was successful. There was no hematoma expansion nor re-bleed acutely nor at multiple interim follow-ups up to a year after the event. This case demonstrated good long-term outcomes but the pulmonary embolism occurred 18 days after the diagnosis of ICH and, therefore, an acceptable risk to treat with systemic anticoagulation [6,7].

Treating a pulmonary embolism occurring within 14 days of an ICH remains a clinical challenge without clear guidelines. This present case report discusses the treatment of a PE diagnosed just 10 days after an ICH. The chosen treatment modality was a percutaneous suction thrombectomy and the outcome was without hematoma expansion nor re-bleed. The aim of this report is to highlight the potential for catheter-directed thrombectomy as a safe means to decrease pulmonary artery clot burden in patients with concomitant ICH.

## Case presentation

**Figure 1:**
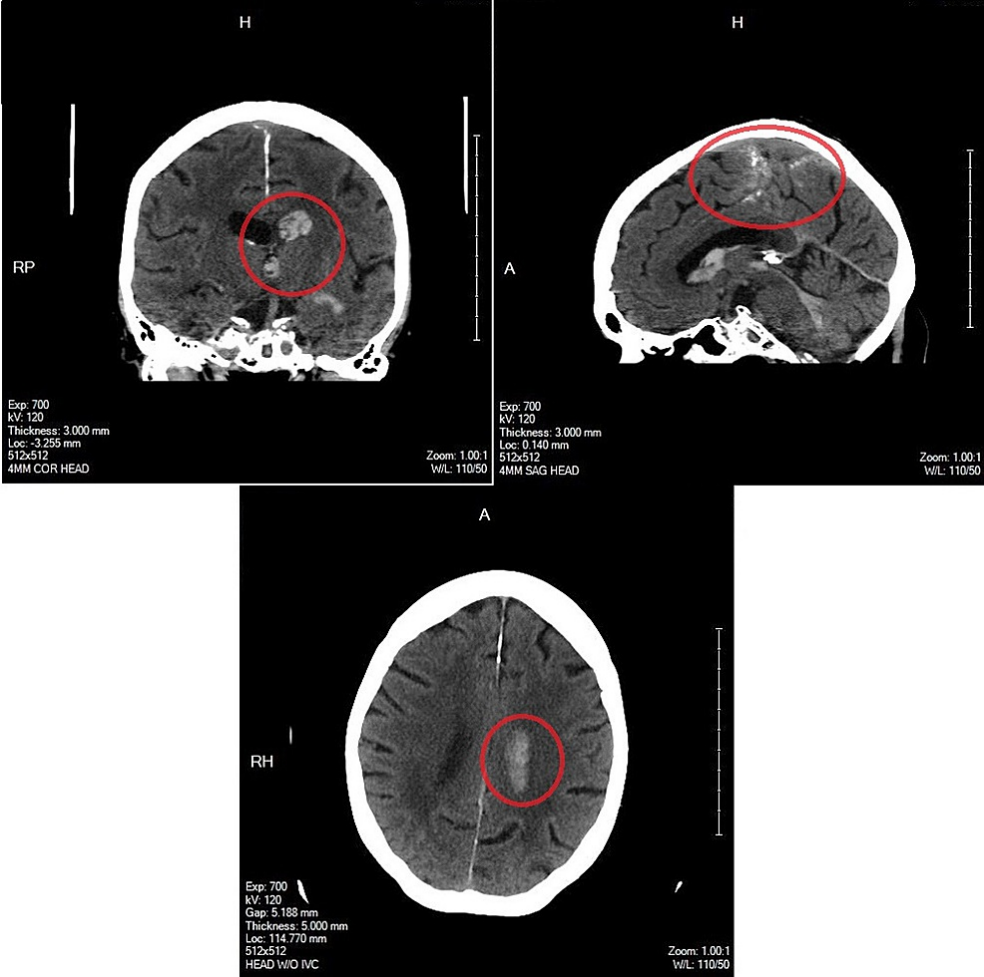
Axial CT (bottom) with sagittal (right) and coronal (left) multiplanar reformatting demonstrating hemorrhage in the bilateral lateral ventricles, third ventricle, and fourth ventricle. There is a tiny focus of hemorrhage in the periventricular white matter adjacent to the high convexity left lateral ventricle. Also seen is mild dilatation of the lateral ventricles.

An 80-year-old female presented to an external hospital with complaints of dizziness, headache, nausea, and vomiting of abrupt onset one hour prior to arrival. Computed tomography (CT) of the head with CT Angiography (CTA) of the head and neck was performed and demonstrated intracerebral hemorrhage (ICH); diffusely intraventricular most prominently within the left lateral ventricle (Figure 1). The patient’s past medical history is significant for systemic hypertension, non-insulin-dependent type 2 diabetes mellitus, chronic obstructive pulmonary disease, and permanent atrial fibrillation anticoagulated with rivaroxaban. Prothrombin complex concentrate was administered for the reversal of her anticoagulation and the patient was transferred to Portsmouth Regional Hospital for neurosurgical evaluation and management. Neurosurgery assessed medical management to be appropriate for this patient and she was admitted to the intensive care unit (ICU). Neurology was consulted and they recommended diagnostic cerebral angiography with neuro-interventional radiology. However, the patient voiced a preference for deferment of any invasive procedures that would not reverse an underlying medical condition. She was to consider this testing in the future in the outpatient setting. Magnetic resonance imaging (MRI) of the brain with and without contrast was recommended to further evaluate her bleed in the absence of this testing. Her ICU course was complicated by hypertensive urgency managed with a nicardipine drip. She also experienced atrial fibrillation with rapid ventricular response managed with a diltiazem drip. The CT head 48 hours after presentation revealed stable parenchymal and intraventricular hemorrhage. The patient’s level of care was de-escalated to the medical wards and the patient was transferred with blood pressure controlled with titratable medication. She would continue to have a rapid ventricular response.

**Figure 2:**
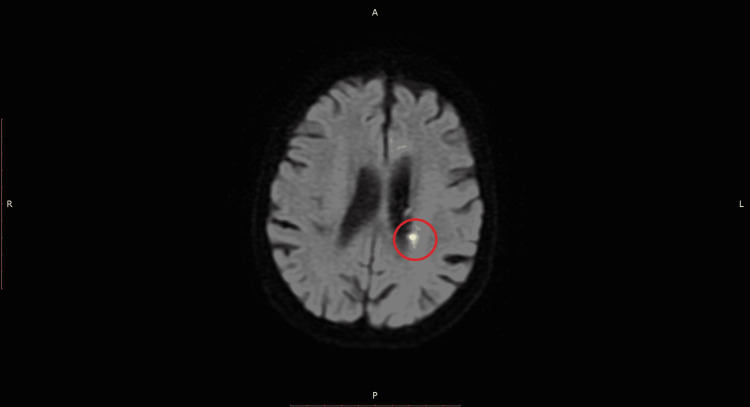
Axial diffusion-weighted MRI of the head/brain WWO IV contrast demonstrating a small focus of diffusion restriction within the white matter abutting the left lateral ventricle consistent with acute infarction.

**Figure 3:**
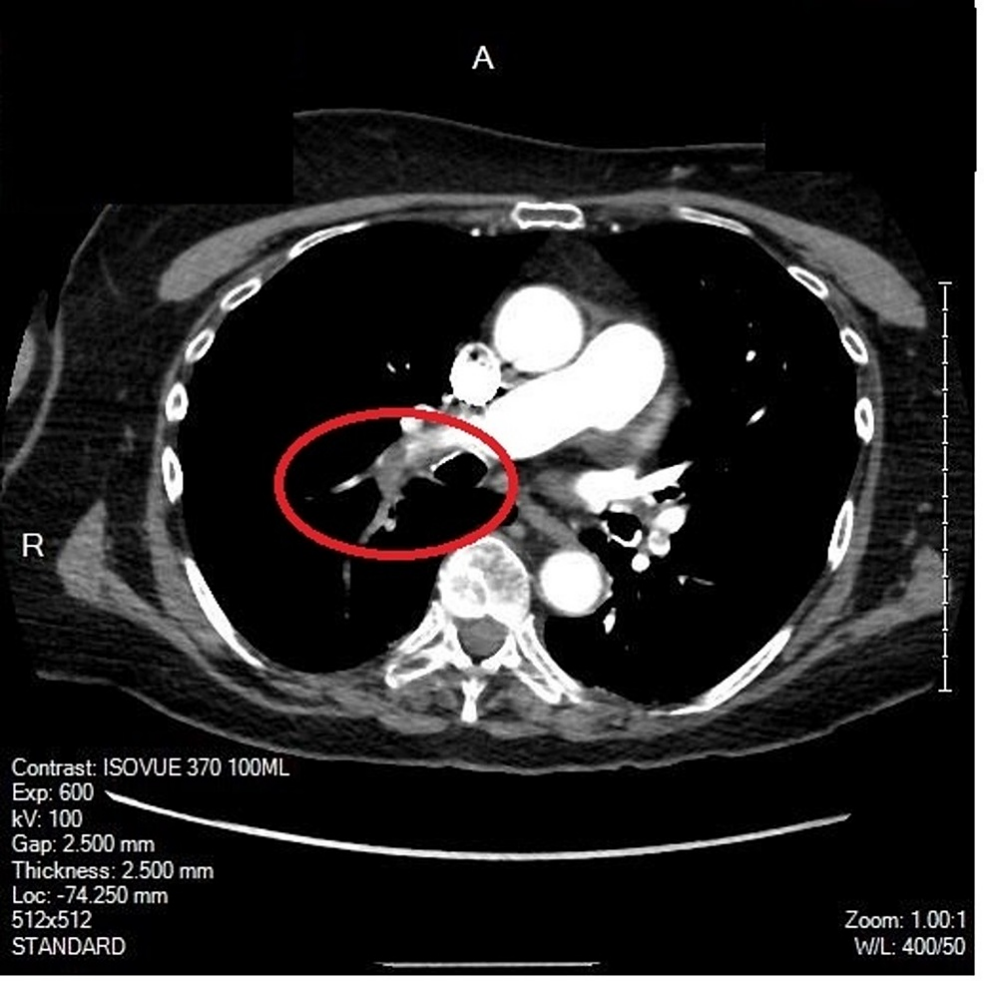
Axial helical CT performed following 75 mL of Isovue-370 IV contrast with multiplanar reconstructions demonstrating filling defects in the distal right main pulmonary artery extending into the lobar branches of the upper and lower lobes and subsegmental branches of the right middle lobe. There is a filling defect in the distal left main pulmonary artery extending into the lobar branches of the upper and lower lobes. No disproportionate right ventricular dilation.

The MRI of the brain (Figure 2) suggested that the cause of her hemorrhage was reperfusion injury after a small acute infarction in the left internal capsule in the setting of anticoagulant use. The patient remained relatively stable until hospital day nine. At that time, on intermediate care, the patient was found in hypoxemic respiratory failure requiring oxygen support with low flow nasal cannula as high as 5 L. In the days leading up to this acute hypoxemia, the patient had been started on ceftriaxone and azithromycin due to the concerns for pneumonia in the setting of leukocytosis, hypoxemia requiring a 2 L low flow nasal cannula, and nonspecific retrocardiac opacity on chest X-ray. Antibiotic therapy was escalated to cefepime and vancomycin due to the acute worsening of her hypoxemia. Azithromycin was discontinued post negative urine antigen tests for *Streptococcus pneumoniae *and *Legionella pneumophila*. The CTA of the chest was ordered with concern for pulmonary embolism. This testing would demonstrate bilateral lobar, segmental, and subsegmental pulmonary emboli (Figure 3). No evidence of right heart strain on CTA but high sensitivity troponin was elevated to 249.1 ng/L. Subsequent ultrasonography of her lower extremities was without deep vein thrombosis bilaterally. This finding would come 10 days after her diagnosis of ICH.

**Figure 4:**
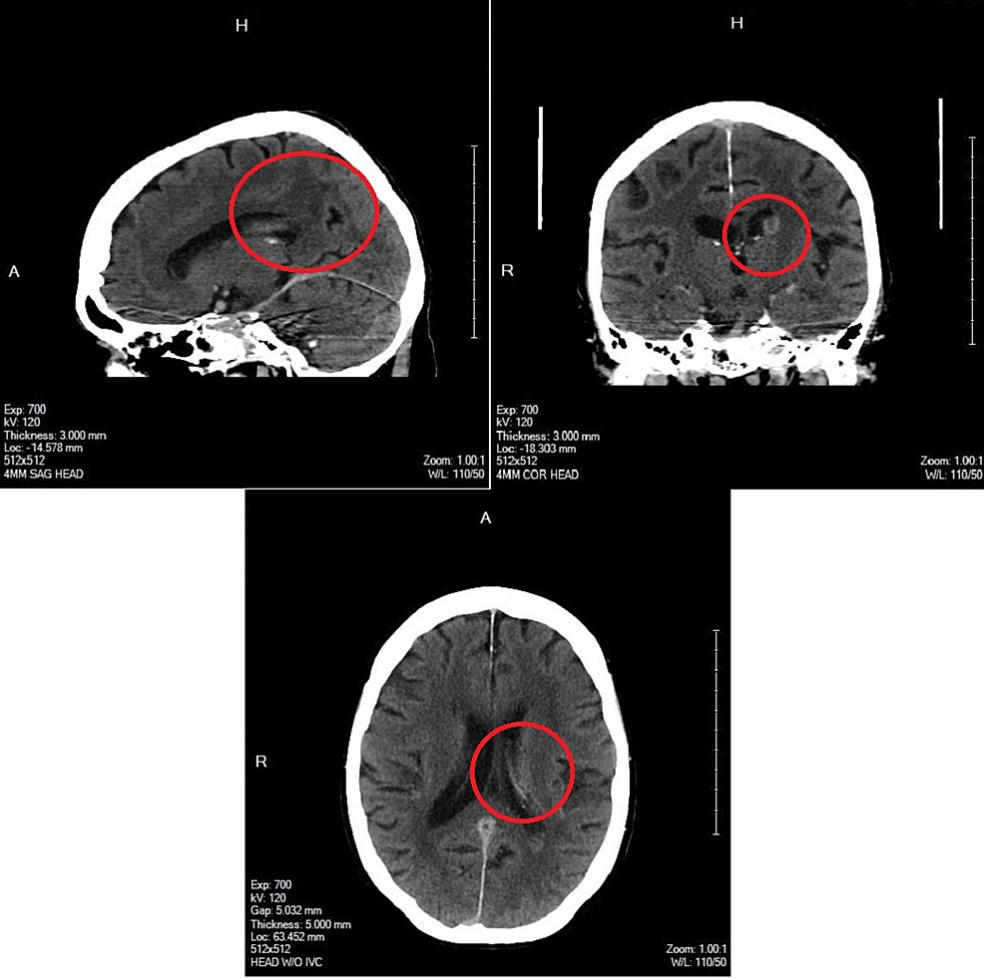
Axial CT (bottom) of the head without contrast, including sagittal (left) and coronal (right) multiplanar reformatted images, demonstrated interval evolution and continued resolution of the interventricular hemorrhage. The majority of the intraventricular blood is hypoattenuating to normal brain tissue and has largely resolved. Blood within the third and fourth ventricles is less conspicuous compared to the prior MRI. There is a small focus on intraparenchymal hemorrhage that remains just superior to the left basal ganglia which are evolving but not enlarging. Lateral ventricles have decreased in size.

An interdisciplinary evaluation was conducted between hospitalist medicine, neurology, neurosurgery, and interventional radiology. Head CT was repeated and demonstrated continued evolution of the ICH without expansion nor new hemorrhage identified (Figure 4). Neurology assessed that systemic anticoagulation would be too high risk to consider at this juncture. Interventional radiology was consulted on the case. The patient was normotensive with positive heart enzymes but no evidence of right heart strain on the CT scan nor electrocardiogram, and therefore assessed to have submassive, low-intermediate risk PE. Echocardiography was not performed. However, given her persistent heart rates into the 120s in the setting of concomitant atrial fibrillation and persistent oxygen desaturations into the 80s while on a 5 L nasal cannula, there was a concern for eventual hemodynamic decompensation. Her pulmonary embolism severity index (PESI) was assessed as class IV, high risk with 4.0% to 11.4% 30-day mortality, given her heart rates and oxygen saturations. A suction thrombectomy was proposed. This would require therapeutic anticoagulation but only for the two-hour duration of the procedure. An inferior vena cava (IVC) filter would also be placed. The proposed treatment was discussed with neurosurgery who assessed that the risk of not treating outweighed the risk of expansion of her ICH in the brief period of anticoagulation anticipated. A successful suction thrombectomy was performed on hospital day 11. Around 150 units of heparin were administered during the procedure. Pulmonary artery pressures improved from 61/25 mmHg pre-procedure to 47/27 mmHg post-procedure. The IVC filter was placed without complication.

No new neurologic deficits were appreciated post-procedure. The patient’s heart rate remained elevated but improved. Blood pressure remained controlled. The patient was weaned off oxygen to room air. Neurosurgery assessed the patient to be of acceptable risk for discharge and deferred anticoagulation until repeat CT head six weeks after discharge. The patient was discharged on day 14 of hospitalization. The patient was lost to follow-up. It is unknown whether she had her follow-up CT performed or a diagnostic cerebral angiogram. 

## Discussion

Percutaneous mechanical intervention is a diverse and emerging means to disrupt and aspirate an obstructing thrombus or embolus [13]. Aspiration or suction thrombectomy, the method used in this case, can be employed via a handful of proprietary devices and techniques [14-18]. In general, a catheter system is used to engage the clot and then a negative pressure vacuum is created to remove the clot [13]. Interventional strategies have lower associated bleeding risks than the standby for PE treatment and systemic anticoagulation. Despite this benefit, there are few examples in the literature of its utilization in the management of PE in the setting of ICH. This is likely owing to the infrequency of this vexing clinical scenario. This case demonstrates that it is a safe and effective option to manage a submassive PE in the setting of ICH. Treating PE in the setting of ICH is without clear guidelines. The appropriate treatment modality is reliant upon the clinical judgment and the individual details of each case. In this case, a high PESI score with imaging demonstrating a stable hematoma without evidence of new blood and a clear source of the original bleed with a low likelihood of re-bleed (ischemic stroke with hemorrhagic conversion) resulted in the decision to use a suction thrombectomy. There is limited documentation of the employment of other techniques in the management of this clinical problem. However, one case did demonstrate that a different technique, modified systemic thrombolysis, was suitable for their given clinical scenario [10].

That case was published in 2018 [10]. The patient presented with a massive pulmonary embolism precipitating a shock state with systolic blood pressures to 70 mmHg and poor right ventricular performance. Systemic thrombolysis would have been indicated were it not for an ICH just 18 days prior. Based on the documentation, it appears the etiology of the ICH was hypertension and, presumptively, without a focus, like an arteriovenous malformation and at high risk for recurrent bleeding. Unfractionated heparin was started given its ability to be converted should bleeding recur. Heparin was infused for 72 hours to prevent additional thrombi and facilitate PE stabilization. The patient was converted to rivaroxaban 15 mg twice daily for three weeks followed by standard dosing of 20 mg daily for one year. Longitudinal imaging did not demonstrate recurrent hemorrhage. The rationale for this regimen was that the patient was greater than 14 days out from their ICH and that rivaroxaban had a safer bleeding profile than vitamin K antagonists. Why a systemic anticoagulation strategy was chosen over an interventional approach was not directly commented upon [10].

Unfortunately, no other examples in the literature could be found where percutaneous mechanical intervention was employed to treat a PE in the setting of ICH to compare the clinical substrates that permit the utilization of this technique. There are cases, however, where catheter-directed techniques were employed in the treatment of a PE only for the outcome to be a new onset ICH [19,20]. These cases highlight that irrespective of the technique in treating PE, none completely mitigates the risk of bleeding. More research is needed to develop consistent evidence-based guidelines for this clinical challenge.

## Conclusions

Pulmonary embolism in the setting of ICH is a potentially fatal combination. Suction thrombectomy may be a safe and effective method for managing PE in the setting of ICH. This present case demonstrated its safety and efficacy in the acute phase. Unfortunately, the patient was lost to follow-up and so longitudinal imaging and morbidity/mortality outcomes are lacking. This case by no means suggests that suction thrombectomy be the standard of care for the management of PE in the setting of ICH. As reported, an interventional approach is not without its hazards. Modified systemic thrombolysis may be an alternative approach if specific clinical criteria are met. Using an interventional approach in this case and a modified systemic thrombolysis approach in a previous case thankfully were successful within the contexts of the specific clinical scenarios in which they were employed. To the author's knowledge, there are no comparative studies to determine the best practice for treating PE in the setting of ICH. Furthermore, there are very few reported cases involving the treatment of this scenario to guide physicians. In conclusion, additional cases and trials are needed to establish a guideline-directed therapy for treating PE in the setting of ICH. This is most relevant in the cases of PE that occur within 14 days of an ICH.
